# Synthesis and Properties of Thermosets from Tung Oil and Furfuryl Methacrylate

**DOI:** 10.3390/polym12020258

**Published:** 2020-01-22

**Authors:** Sunanda Sain, Dan Åkesson, Mikael Skrifvars

**Affiliations:** Swedish Centre for Resource Recovery, University of Borås, SE-501 90 Borås, Sweden; mikael.skrifvars@hb.se

**Keywords:** thermoset, cross-linking, Diels–Alder reaction, free radical polymerisation

## Abstract

This work focuses on the development of cross-linked polymer from a highly unsaturated vegetable oil, tung oil (TO) and a bio-based acrylate, furfuryl methacrylate (FMA). The presence of a high degree of unsaturated carbon-carbon bonding in TO makes it a suitable precursor for polymer synthesis. Using this advantage of TO, in this work, we have synthesised a cross-linked polymer from TO and FMA through free radical polymerisation followed by Diels–Alder (DA) reaction. Successful incorporation of both of the raw materials and the two chemical reactions was shown using Fourier-transform infrared (FTIR) and Raman spectroscopy. The development of cross-linked structure was analysed through thermogravimetric analysis (TGA) and dynamic mechanical analysis (DMA).

## 1. Introduction

**Figure 1:**
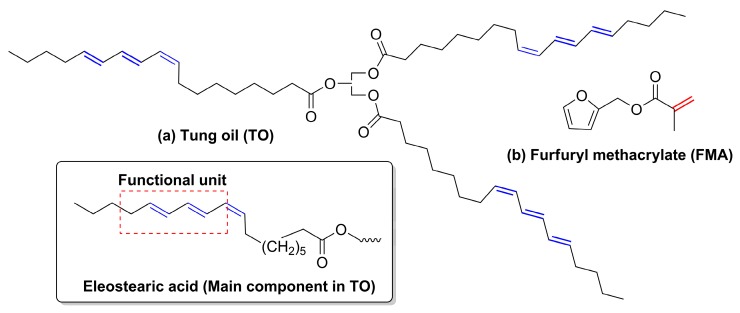
The chemical structure of vegetable oil and acrylate monomer: (**a**) tung oil (TO) and (**b**) furfuryl methacrylate (FMA).

Depletion of fossil fuels and growing concerns towards environmental issues spurred our interest in developing bio-based materials, which can reduce our dependency on petroleum-based polymers. In this context, vegetable oils are important raw materials with different functionalities for development of new polymers. Major advantages of this bio-based resource include low cost, availability, renewability and biodegradability. Vegetable oil-derived polymeric materials with good thermomechanical and excellent damping properties will help to replace petroleum-based products in the near future [1,2]. Highly cross-linked thermoset-type materials can be produced from fatty acid unsaturation [1]. Soybean oil, castor oil and linseed oil (among others) have widely been used for polymer synthesis [3,4,5]. Compared to other vegetable oils, tung oil (TO), which is obtained from seeds of the tung tree, has remained relatively underexplored as a thermoset precursor [6,7]. This oil is mostly used in paint and varnish formulations because of its fast drying property [8]. It is a key precursor for synthesis of new polymers since it has a high degree of unsaturation in its structure (Figure 1a).

The main component of tung oil is α-eleostearic acid (*cis*-9, *trans*-11, *trans*-13-octadecatrienoic acid—Figure 1a), which is responsible for the interesting properties of this oil by providing long hydrocarbon chains and conjugated triene structures [9]. So far, cationic, thermal, emulsion, free radical and co-polymerisation techniques have been investigated with tung oil, styrene, divinyl benzene and various acrylate monomers to manufacture a wide variety of products ranging from soft rubbery to highly cross-linked polymers [8,10,11,12]. Its highly unsaturated structure makes TO a good material for the Diels–Alder (DA) reaction in the presence of suitable dienophiles such as 1,6-hexanediol-diacrylate and 1,4 butanediol-diacrylate [12]. UV-curable tung oil-acrylate resin has been synthesised through DA reaction [13]. Thanamongkollit and Soucek synthesised acrylate-modified tung oil alkyd resin by the same reaction [14].

To date, petroleum-based acrylates or other vinyl monomers have been investigated for co-polymerisation with tung oil, while, in the present work, we focused on incorporating bio-based furfuryl methacrylate (FMA (Figure 1b)). Biomass-derived FMA is another interesting monomer in the synthesis of speciality bio-based materials [15]. Free radical polymerisation is a widely used process in polymer synthesis, and, according to previous reports, conventional free radical polymerisation of FMA yields cross-linked polymer gel [16,17]. Different attempts have been made to avoid gelation during polymerisation of FMA via atom transfer radical polymerisation [17,18], organocatalytic polymerisation [15], and by synthesising the copolymers using styrene, acrylonitrile, etc. [16,19].

In this study, we investigated the effect of free radical polymerisation of FMA in the presence of highly reactive TO with long-chain hydrocarbon backbones. The objective was to exploit the reactivity of the conjugated trienes in TO *via* two steps to fabricate a cross-linked network: first, free radical polymerisation with the acrylate group of FMA, followed by DA reaction between the furfuryl moiety of FMA and the remaining unreacted double bonds of TO. Importantly, the long hydrocarbon chains in TO would control the gel formation between FMA molecules.

## 2. Materials and Methods

Furfuryl methacrylate (FMA, 97%, containing monomethyl ether hydroquinone as inhibitor), tung oil (TO), Luperox^®^ A75 benzoyl peroxide (BPO, 75%) and alumina (Al_2_O_3_) were purchased from Sigma Aldrich, Stockholm, Sweden. Toluene, dehydrated (≤0.005% H_2_O), ≥99.8% (analytical reagent for synthesis), was used as a solvent and was obtained from VWR Chemicals (VWR International AB, Stockholm, Sweden). FMA was made inhibitor-free before the polymerisation reaction by passing it through a basic alumina column. All the reagents used in this study were of reagent grade.

Reaction mixtures were prepared with various monomer concentrations, such as FMA:TO ratios of 1:1, 2:3 and 3:7 (wt %). BPO (3 wt % w.r.t. monomer weight) was added to each set as the initiator. The polymerisation reaction was carried out in round-bottomed flask fitted with a reflux condenser. N_2_ gas was passed through the reaction vessel for 15 min before the start of the reaction. The temperature was maintained at 80 °C for 4 h followed by 120 °C for 2 h. After completion of the reaction, white crude powder samples were obtained. Pure polyfurfuryl methacrylate (PFMA) was synthesised by free radical polymerisation using the same reaction conditions. The detailed characterisation of PFMA is described in the Supplementary Materials. Toluene was used to wash the crude product to remove unreacted monomers and impurities. The sample codes and compositions are given in Table 1. Percentage yield was calculated based on the total weight of the raw materials (*w*_r_) and the total weight of the product (*w*_p_) obtained after the polymerisation, using the following formula (1): %yield = (*w*_p_/*w*_r_) × 100.

The solubility of the powder samples was verified using different solvents such as chloroform, tetrahydrofuran, toluene and n-heptane.

The powder samples, coded as F50-T50, F40-T60 and F30-T70, were then processed in a compression moulding machine (Rondol Kompress 20T; Rondol Technology Ltd., London, UK) with a pressure of 125 kPa to produce polymer films for mechanical characterisation. The dimensions of the square mould were 20 cm × 20 cm and the film thickness obtained was 1 mm. The samples were heated at 140 °C for 4 h to make the films. The film sample codes used in this work were F50-T50-F, F40-T60-F and F30-T70-F.

Attenuated total reflectance Fourier-transform infrared spectroscopy (ATR-FTIR, Nicolet is10, Thermo Fisher Scientific, Waltham, MA, USA) was used to check the free radical polymerisation between FMA and TO. To ensure the participation of double bonds in tung oil in the reactions, Raman spectra were recorded with an Ocean Optics Raman spectrometer (QEP00920, Ostfildern, Germany) using a laser operating at 785 nm, with a slit width of 50 µm. Differential scanning calorimetry (DSC Q1000, TA instruments, Newcastle, UK) was used to study the curing behaviour of the prepared polymers, and to obtain the glass transition temperature (*T*_g_) of the films. The experiments were carried out in a nitrogen environment at 25–250 °C, and at a heating rate of 10 °C/min. The first heating scans for all the samples were recorded. Thermogravimetric analysis (TGA Q1000, TA instruments, Newcastle, UK) was performed in a nitrogen environment with a temperature range of 30–600 °C and a heating rate of 10 °C/min, to assess the thermal stability of the prepared polymers. The viscoelastic properties of the polymer films were investigated by dynamic mechanical analysis (DMA) (DMAQ800, TA instruments, Newcastle, UK) in multi-frequency strain mode. The frequency was 1 Hz and the temperature range was 25–180 °C, with a heating rate of 3 °C/min. The morphology of the prepared polymer films was examined with scanning electron microscopy using a TESCAN MIRA3 FEG-SEM with an SE detector (Everhart–Thornley type, Brno, Czech Republic) and an accelerating voltage of 5 kV. The images were taken at a magnification of 10,000×. Optical microscopic images of the film samples were recorded using a Nikon SMZ800 optical microscope (Amsterdam, Netherlands) at a magnification of 3.0×. The Soxhlet extraction was performed to collect the soluble portion from film samples. One gram of each sample (F50-T50-F, F40-T60-F and F30-T70-F) was refluxed with toluene for 12 h. The soluble portion was then collected separately and analysed by FTIR and NMR. ^1^H NMR and ^13^C NMR were performed using a Bruker DPX 400 spectrometer, Coventry, UK and toluene-d_8_ was used as the solvent for NMR analysis. The insoluble portion was dried overnight in a vacuum oven and the yield was calculated using formula (1).

## 3. Results and Discussion

**Figure 2:**
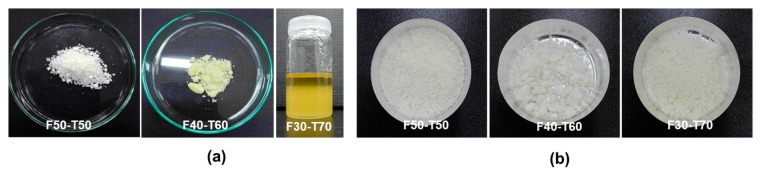
(**a**) As-synthesized samples after the free radical polymerisation, and the (**b**) same samples after washing with toluene.

Tung oil (TO) and furfuryl methacrylate (FMA) were free radically polymerised and white powder-like samples were obtained (F50-T50, F40-T60 and F30-T70). The powder samples were then hot-pressed to obtain the film samples, coded as F50-T50-F, F40-T60-F and F30-T70-F. Solid powder and film samples were analysed using spectroscopic, thermal and microscopic techniques. First, the yield was calculated after the free radical polymerisation reaction (Table 1), and it was observed that with increasing TO content the unreacted portion of the monomer increased (Figure 2a,b). The amount of yield just after the reaction, and after washing, varied significantly. This indicated that a specific proportion of the monomer was taking part in polymer formation and the rest of the monomer remained unreacted. FTIR analysis of the samples was done to confirm the incorporation of both monomers during the polymerisation reaction.

**Figure 3:**
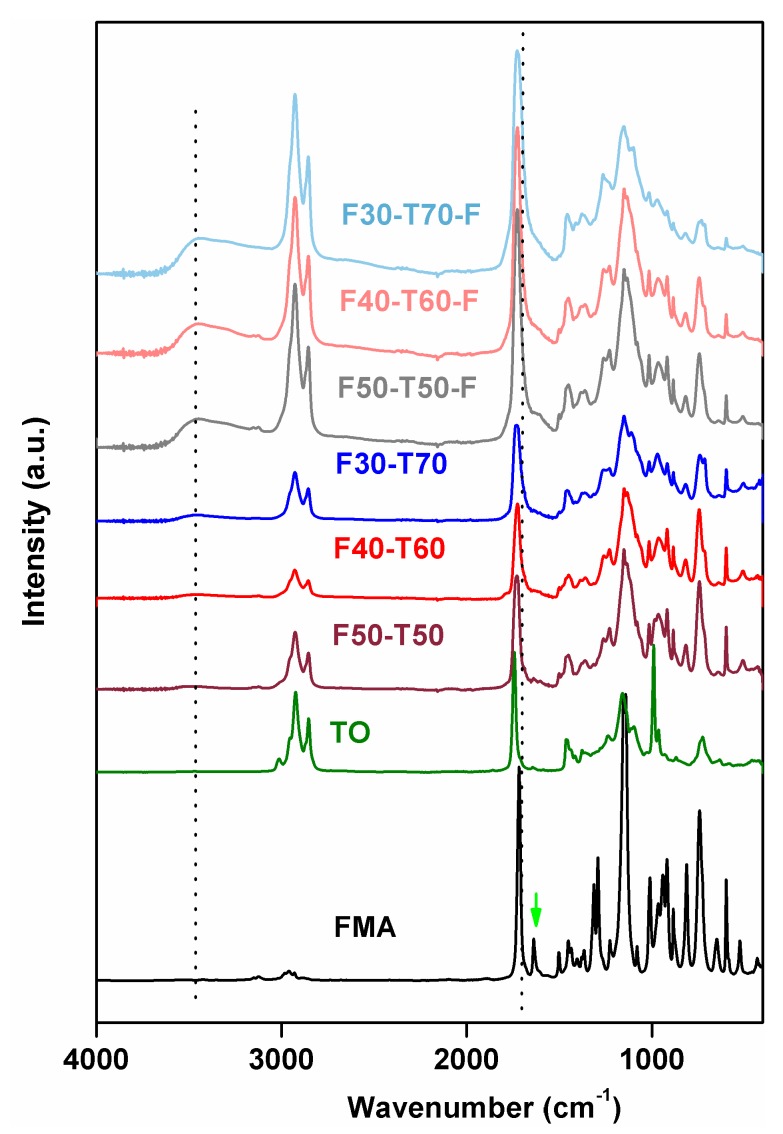
FTIR (Fourier-transform infrared) plots of powder and film samples (dotted line indicates peaks at 3469 and 1716 cm^−1^; green arrow indicates peak at 1634 cm^−1^).

The FTIR results (Figure 3) showed that typical acrylate C=C stretching from FMA at 1637 cm^−1^ had completely disappeared in prepared polymers. Likewise, the characteristic peaks at 991 and 964 cm^−1^ for C–H wagging vibrations, due to the presence of conjugated *cis* and *trans* double bonds in TO, also disappeared. This confirmed the participation of acrylate groups from FMA in free radical polymerisation with conjugated double bonds from TO. In addition, both FMA and TO contained ester stretching at 1716 cm^−1^ (for methacrylate groups in FMA) and 1742 cm^−1^ (for triglyceride ester stretching in TO), respectively. In the polymer samples, these peaks merged and appeared as broad signals at 1730 cm^−1^. This indicated the presence of both types of carbonyl environments in the polymers. The peaks at 1501, 1153 and 1012 cm^−1^ corresponded to furan ring stretching, –C–O–C– ether stretching for furan groups and furan ring breathing in FMA, respectively [17,20]. These peaks for furan rings remained intact in polymers, which explained that the furan ring itself did not take part in free radical polymerisation due to its stable aromatic ring structure. Small –C–H stretching vibration at 3012 cm^−1^, associated with C=C in TO, was also absent from all powder and film samples [6,21]. Since both the raw materials, FMA and TO, are hydrophobic in nature, there is little possibility of moisture absorption in the developed polymer product. Thus, the presence of broad peaks at 3469 cm^−1^ in F50-T50-F, F40-T60-F and F30-T70-F could be attributed to the incorporation of oxygen into the unreacted double bonds in TO, i.e., oxidative curing happened during film formation in the presence of oxygen. This kind of oxidative curing was observed in co-polymer films obtained from tung oil and di-acrylates [12].

**Figure 4:**
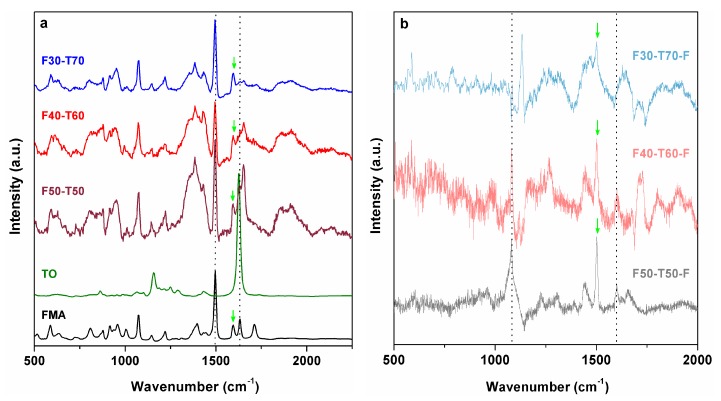
Raman spectra of (**a**) powder samples (dotted lines indicate the peaks at 1623 and 1497 cm^−1^; green arrow indicates the peak at 1593 cm^−1^) and (**b**) film samples (dotted lines indicate the peaks at 1600 and 1076 cm^−1^; green arrow indicates the peak at 1500 cm^−1^).

To confirm the complete participation of acrylate groups in polymerisation and to assess whether there was any unsaturation formed through DA reactions, Raman spectra were recorded for all samples (Figure 4). In polymer (powder) samples, the C=C stretching shifted to 1651 cm^−1^ and the signal had broadened, which may have been due to overlapping of close vibrations from FMA and TO. The furan ring C=C stretching at 1490 and 1594 cm^−1^ in FMA remained unaltered in powder polymers (F50-T50, F40-T60 and F30-T70), whereas these peaks were absent in the film samples. New signals appeared at 1600–1650 cm^−1^ in the film samples (1603 cm^−1^ for F50-T50-F and F40-T60-F and 1640 cm^−1^ for F30-T70-F), which may have been due to the formation of new double bonds via DA reactions between furan moieties and unreacted double bonds in TO during film formation [22]. Participation of furan moieties of FMA in DA reaction has previously been reported by Geitner et al. [22] and by Feng et al. [15]. The weak intensity and broad nature of these signals indicated the probable oxidative curing of the remaining TO double bonds and overlapping of close vibrations, respectively [23]. The presence of a furan ring stretching at 1503 cm^−1^ in the film samples indicated that not all of the furan moieties participated in cycloaddition reactions with TO [22].

**Figure 5:**
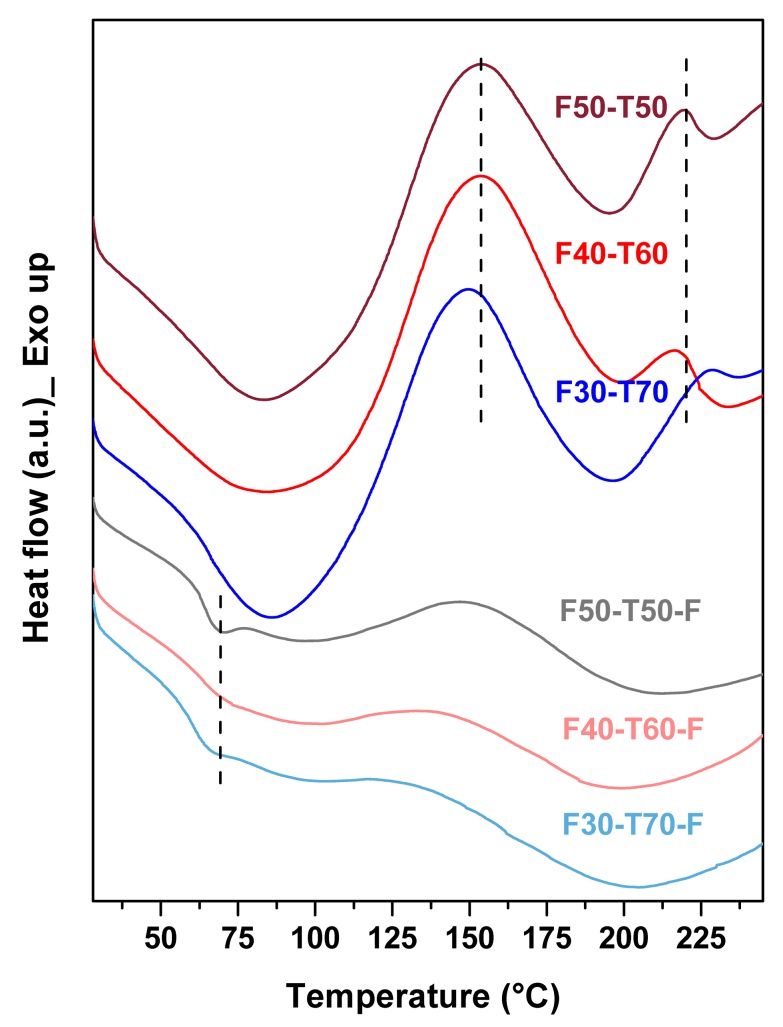
DSC (differential scanning calorimetry) plots of powder and film samples (first heating scans): dash lines in powder samples indicate the exothermic transitions at 153 and 220 °C and the dash line in film samples indicate *T*_g_ at 69 °C.

DSC was used to obtain a better understanding of the curing reactions that took place in the synthesised polymers. From DSC (Figure 5), it was found that F50-T50, F40-T60 and F30-T70 samples showed two exothermic transitions in the temperature range between 150–175 °C and 200–225 °C [1]. This indicated that the samples were not cured properly, i.e., that some double bonds in tung oil and furan rings were still free to react via the DA mechanism, and that with increasing temperature these bonds were taking part in chemical reactions [23]. Two separate exothermic transition regions in the polymers suggested that the extent of reaction between two components varied with different composition of the polymers. Thus, the films had been produced to post cure the samples. In film samples, no sharp exothermic transition was observed, and this result was consistent with the change in Raman spectra from powder polymer to film samples. F50-T50-F, F40-T60-F and F30-T70-F showed small transitions at around 55–65 °C. This is similar to the glass transition temperature (*T*_g_) of polyfurfuryl methacrylate (PFMA) [15]. The DSC curve of free radically polymerised PFMA is shown in the Supplementary Materials (Figure S2). This suggested that there may have been a few molecules of FMA that self-polymerised among themselves. With increasing amounts of TO, *T*_g_ shifted to a lower temperature; thus, for F50-T50, *T*_g_ was around 69 °C, whereas in F30-T70-F it had shifted to 64 °C. This may have been due to the increase in long hydrocarbon chains, which increased the free volume in the system and made the movement of PFMA chains easier. It should be noted that self-polymerisation of FMA produced PFMA as a minor by-product. Greater reactivity of TO to FMA radicals probably facilitated the formation of cross-linked polymer and suppresses the self-polymerisation of FMA, which would probably require higher activation energy [16,24]. The absence of any melting transition in the film samples confirmed the cross-linked character of the resulting polymers. The presence of endothermic transition in the film samples at around 170–225 °C may have been due to thermal degradation of the materials, which was further confirmed by TGA.

**Figure 6:**
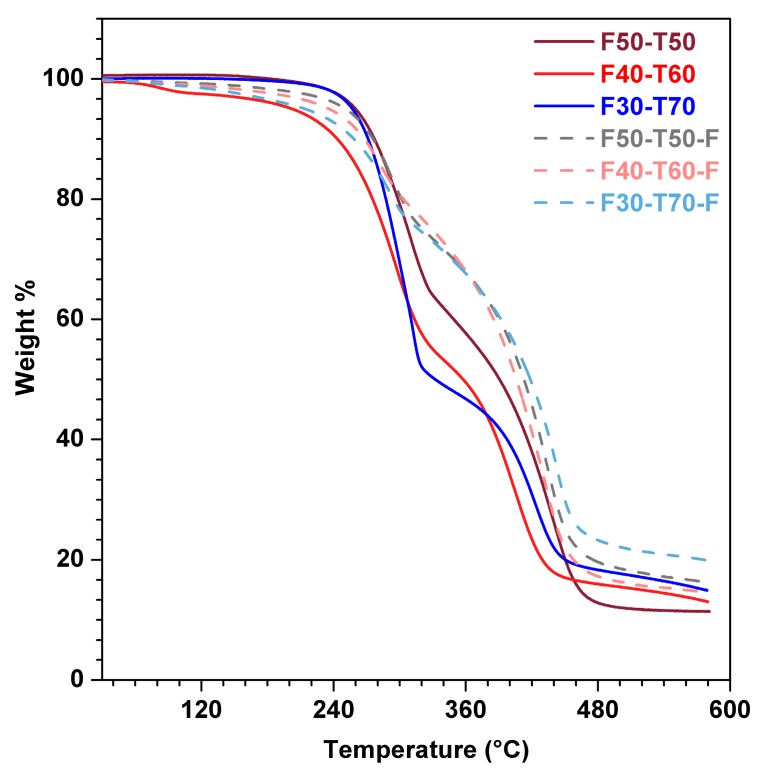
TGA (thermogravimetric analysis) plots of the powder and film samples.

Thermogravimetric analysis (Figure 6) was performed for all the powder samples (F50-T50, F40-T60 and F30-T70) and all the film samples (F50-T50-F, F40-T60-F and F30-T70-F) to assess the thermal stability. This showed that film samples had higher thermal stability than powder solid samples at higher temperatures from 250 °C to 460 °C, which could be attributed to the formation of cross-linked structures due to the participation of furan moieties and also unreacted double bonds of TO. The degradation temperatures at various % weight loss values are given in Table 2. Thermal degradation took place in several stages, which may have been due to the scission of the vinyl bonds and random scission of the polymer chains [15]. The lower thermal stability of the polymer films (F50-T50-F, F40-T60-F and F30-T70-F) than that of the powder materials (F50-T50, F40-T60 and F30-T70) at the lower temperature range (130–240 °C) might be explained by the formation of six-membered ring structures between tung oil and FMA through DA reactions [15].

**Figure 7:**
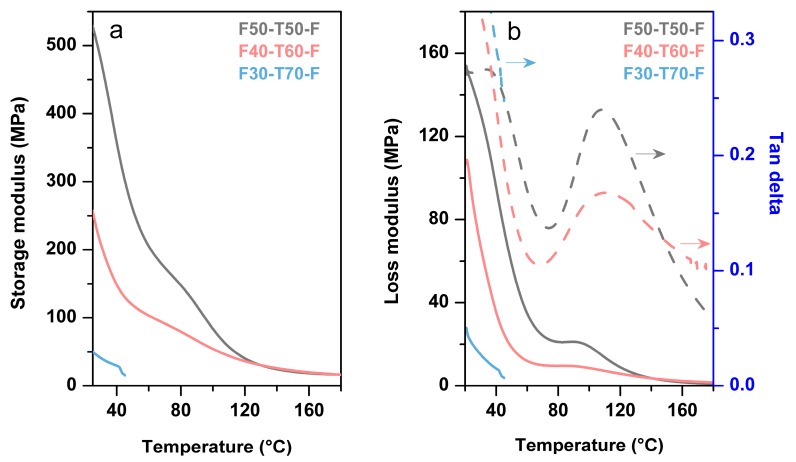
DMA (dynamic mechanical analysis) plots ((**a**) storage modulus and (**b**) loss modulus and tan delta) of film samples F50-T50-F, F40-T60-F and F30-T70-F (In Figure 7b, arrows indicate the tan delta axis).

Figure 7 displays the DMA analysis of the film samples which demonstrates the variation of the storage and loss modulus with temperature. F50-T50-F showed a higher storage modulus (540 MPa) than F40-T60-F (340 MPa) and F30-T70-F (60MPa) at room temperature (Table 2). These values are comparable to the reported storage modulus values of other tung oil polymers [2,9,25]. It was difficult to run F30-T70-F samples, as these samples were too brittle to continue in tension mode, and broke above 45 °C. This might be due to the effect of composition of the polymers. Enhanced TO content increased the soft segment portion of the polymer system due to the long and flexible triglyceride units. This helped in plasticising the network. On the other hand, the number of conjugated double bonds increased per molecule with increasing amounts of TO. This helped in development of more cross-linking sites, which consequently led to rigidity in structure as well as brittleness in the materials. This resulted in polymers with low mechanical properties [2,25]. In Figure 7b, F40-T60-F exhibited broad tan δ transition compared to F50-T50-F, which indicated the presence of a heterogeneous polymer network in the system [14]. The presence of more functional groups in the materials with higher TO content resulted in a mixture of polymer networks with high and low degrees of cross-linking. This led to a broad distribution of mobility of the polymer chains [26]. Moreover, F40-T60-F had a higher *T*_g_ (114 °C) than F50-T50-F (107 °C). This may have been due to the enhanced cross-linking sites, which could overcome the plasticising effect of TO [25]. At a higher temperature range (130–180 °C), storage modulus plots of all samples showed a rubbery plateau with low storage modulus, which confirmed the presence of a stable cross-linked network structure in the prepared polymers. Both F50-T50-F and F40-T60-F samples showed the same cross-link density (~4265 mol m^−3^), as both of the storage modulus plots merged together at a rubbery plateau region. This indicated that a specific proportion of the raw materials took part in cross-linking reactions. Cross-link density was calculated using the formula *E*′ = 3*nRT* (where *E*′ is the storage modulus at the rubbery region, *n* is the cross-linking density, *R* is the universal gas constant and *T* is absolute temperature in K (*T* = *T*_g_ + 25 °C)) [10,27].

**Figure 8:**
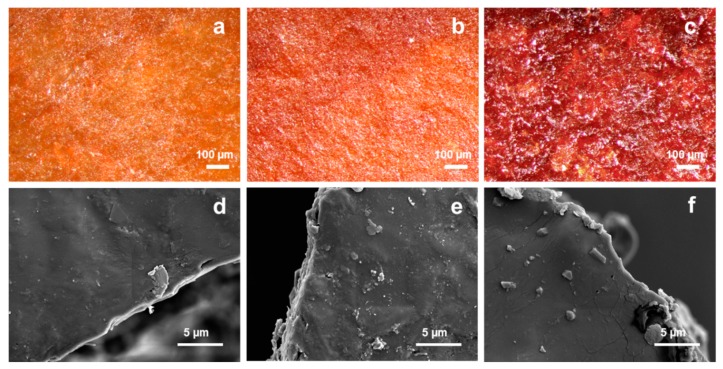
Optical microscopic images of (**a**) F50-T50-F, (**b**) F40-T60-F and (**c**) F30-T70-F; SEM images of (**d**) F50-T50-F, (**e**) F40-T60-F and (**f**) F30-T70-F at 10,000× magnification.

The surfaces of the films were examined using optical microscopy and SEM. Figure 8 shows the optical microscopic images and SEM images of film samples F50-T50-F, F40-T60-F and F30-T70-F. It was found that, with increasing amounts of tung oil in the samples from F50-T50-F to F30-T70-F, the surface became rough and uneven with cracks. With increasing TO content, the number of reactive conjugated double bonds increased. This enhanced the polymerisation rate and the likelihood of oxidative curing of the unreacted double bonds during film formation. As a result of this, the films formed with wrinkled and uneven surfaces with poor mechanical properties, as could be seen in the DMA results.

**Figure 9:**
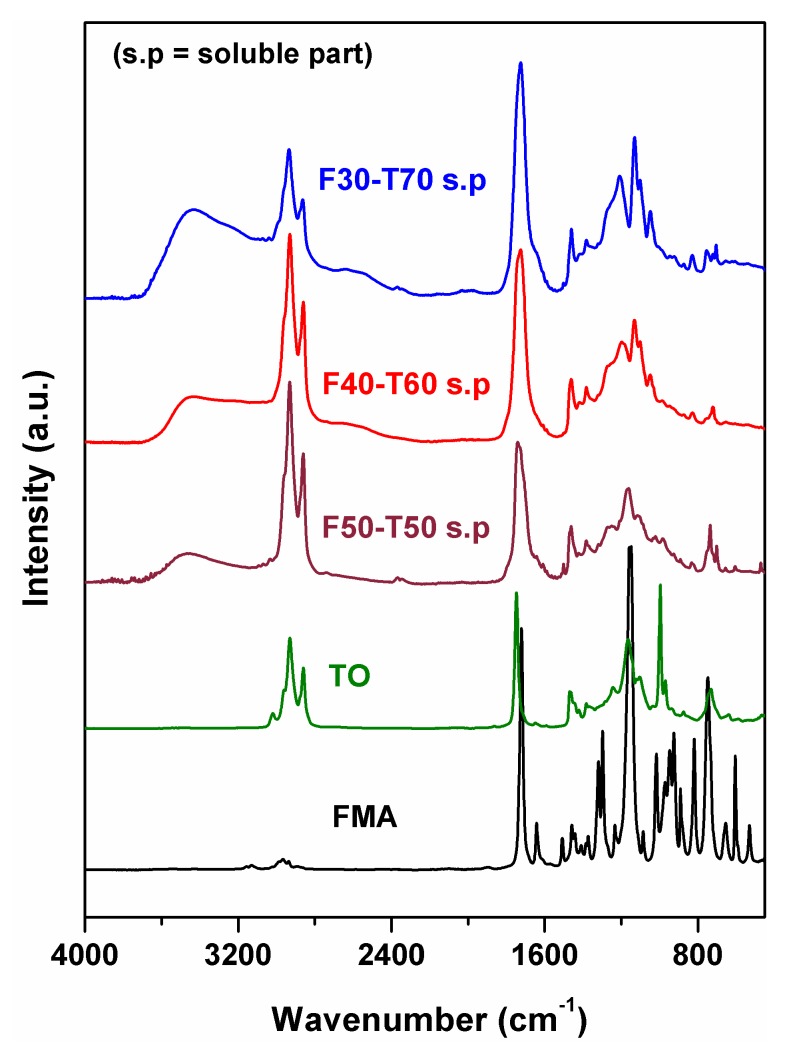
FTIR (Fourier-transform infrared) plots of Soxhlet extracted portions from FMA-TO (furfuryl methacrylate-tung oil) polymers.

It was previously observed from the DMA results that, at high temperature, the synthesised polymeric materials had a stable cross-linked network, and also that the polymers showed poor solubility in common organic solvents. The polymer film samples were therefore Soxhlet extracted with toluene under reflux condition to separate the lower molecular weight oligomers. This Soxhlet extraction of the polymers resulted in a weight loss of the polymer film samples by 12 ± 2%, which means that the rest of the part of the polymer took part in the formation of a cross-linked network structure. The existence of this cross-linked network was observed from storage modulus plot in DMA, as we mentioned before. FTIR plots of Soxhlet recovered portions from FMA-TO polymers revealed the presence of a TO carbonyl environment at 1730 cm^−1^ and sharp –C–H stretching at 2800–3000 cm^−1^ (Figure 9). No C=C stretching from FMA or TO was observed in the spectra. Notably, a broad signal for –O–H stretching appeared at each of the Soxhlet recovered portions from FMA-TO polymers, which could be attributed to the partially reacted TO or TO monoglycerides [28]. This confirmed the presence of TO oligomers in the soluble portions [28].

**Figure 10:**
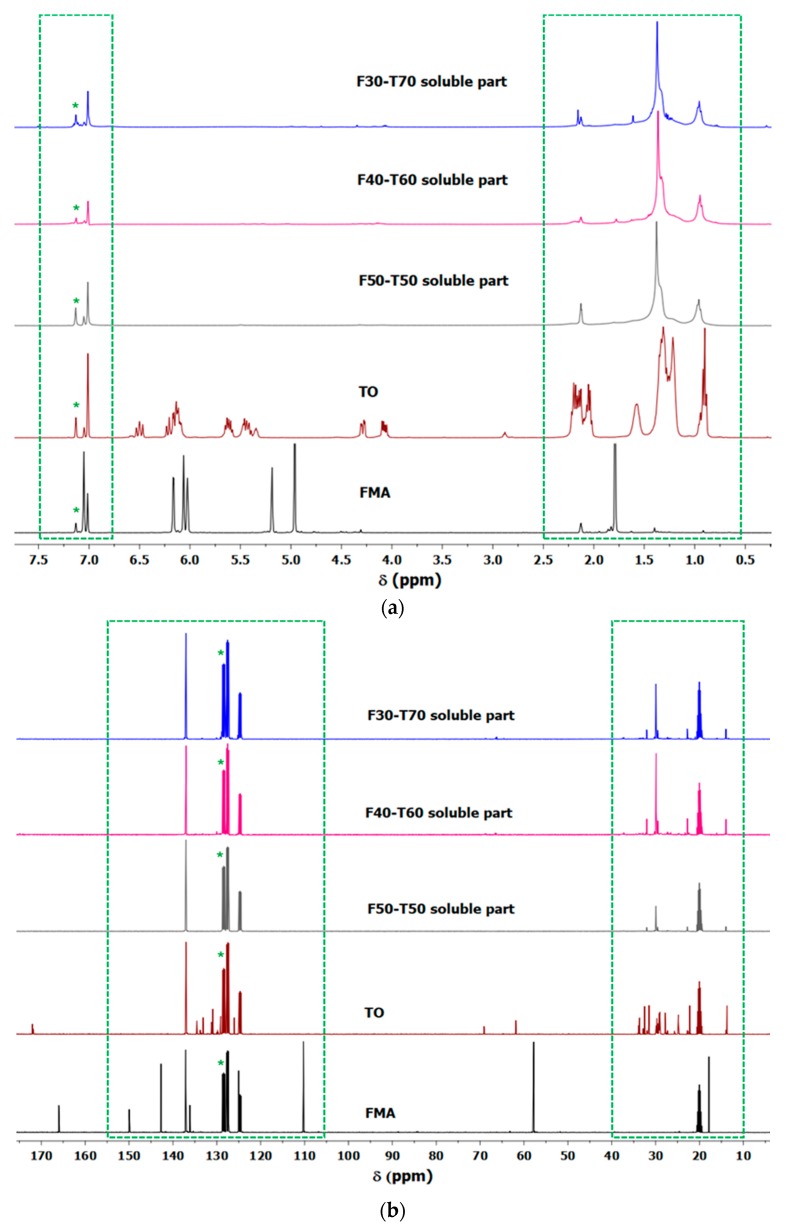
(**a**) ^1^H NMR (nuclear magnetic resonance) signals of Soxhlet extracted portions from FMA-TO polymers (green square shape indicates the differences in signals among the samples in the region δ 0.5–2.5 ppm and δ 6.7–7.5 ppm; * indicates the solvent peak). (**b**) ^13^C NMR signals of Soxhlet extracted portions from FMA-TO polymers (green square shapes indicate the differences in signals among the samples in the region δ 10–40 ppm and δ 105–155 ppm; * indicates the solvent peak).

For further confirmation regarding the Soxhlet extracted portions from synthesised polymers, ^1^H and ^13^C NMR spectra were recorded (Figure 10a,b). In Figure 10a, the proton signals of TO at δ 4.05–4.10 ppm and 4.27–4.31 ppm correspond to glycerol methylene units in TO. The multiplets appearing at δ 5.33–6.59 ppm are attributed to the olefinic protons in TO [2,29]. For FMA, the methylene proton (–CH_2_–OCO–) signal appears as a singlet δ 4.96 ppm, the vinyl and furan-ring protons in the downfield region (δ 6.03–7.05 ppm) and the α-CH_3_ protons at δ 1.79 [30]. The ^13^C NMR spectrum of TO consists of the methylene carbon peaks from glyceride unit at δ 61.9 ppm and 69.3 ppm, unsaturated carbon signals in the range δ 124–137 ppm and the carbonyl from the ester groups at δ 172 ppm [29]. ^13^C NMR spectrum of FMA displays carbonyl peaks at δ 166 ppm, methylene carbon at δ 57.7, α-CH_3_ at δ 18–22 ppm, unsaturated and furan-ring carbons in the range at δ 110–130 ppm [31]. In the soluble portions of FMA-TO polymers, characteristic peaks from TO were present in the 0.5–2.5 ppm region (highlighted with green square shape in Figure 10a). The broad range of peaks in the 5.5–6.5 ppm (highlighted with green square shape in Figure 10a) region suggested a conjugated triene structure for TO, which was absent in soluble portions of FMA-TO polymers [32]. The absence of any characteristic peaks of FMA in the 5–6 ppm region confirmed that the predominant TO-related compounds in the soluble portions of FMA-TO polymers might be TO oligomers [2]. A similar conclusion was reached from ^13^C spectra of FMA-TO polymers, as the TO-related carbon predominated in the 120–150 ppm and 10–40 ppm regions (highlighted with a green square shape in Figure 10b).

**Figure 11:**
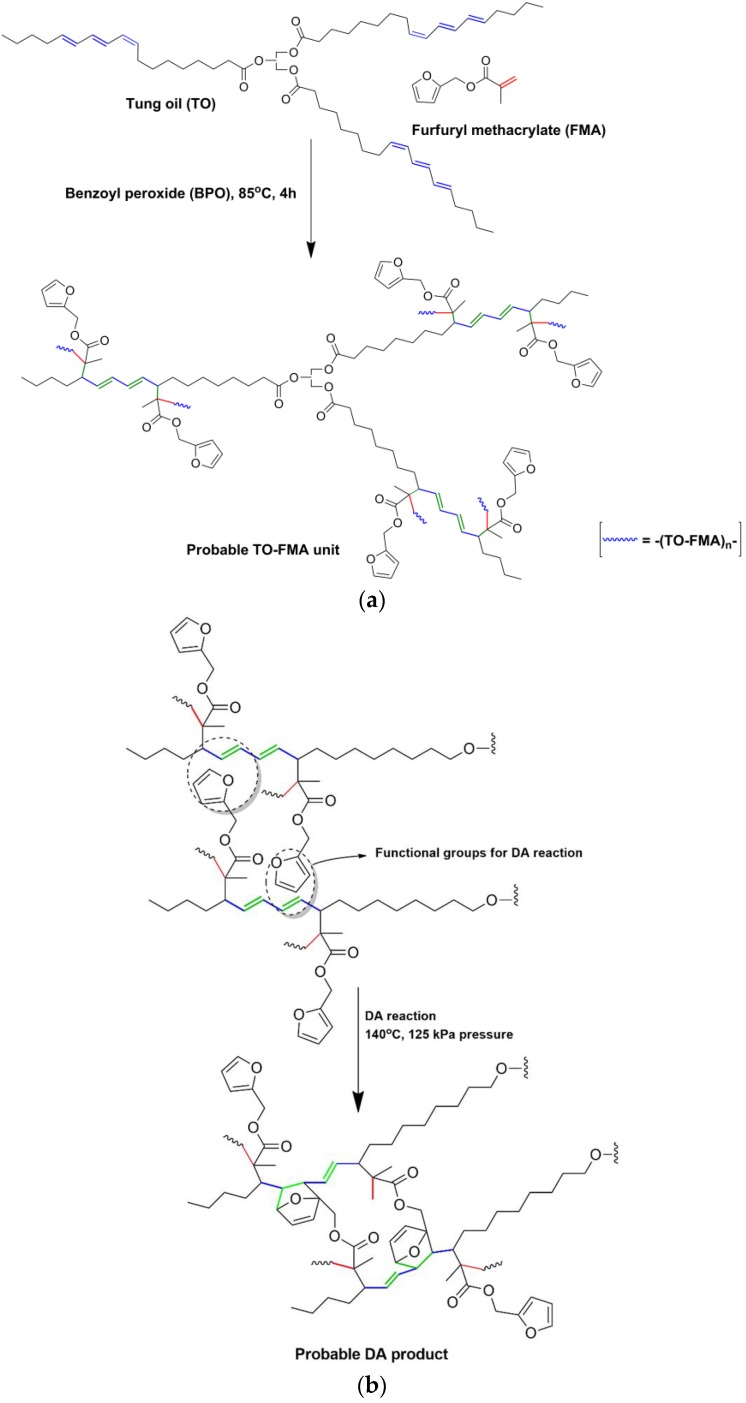
(**a**) Probable reaction scheme for free radical polymerisation. (**b**) Probable reaction scheme for Diels-Alder (DA) reaction.

While TO content increased in the system, a specific proportion of TO took part in cross-linking reactions and the rest contributed to the formation of soluble fragments. As a result, TO-related compounds predominated in Soxhlet soluble portion [1]. Based on the above results and analysis, a probable structure of the polymer was proposed (Figure 11).

According to the published literature, free radical polymerisation of FMA leads to cross-linked gel-like materials [16,17]. In this work, we observed that the presence of TO during free radical polymerisation could help to control the gelation between FMA molecules. Cross-linked polymer networks had been developed in combination with FMA and TO molecules through free radical polymerisation followed by DA reaction. To make sure about this, polyfurfuryl methacrylate was synthesised separately by free radical polymerisation and analysed using FTIR, DSC and TGA to obtain better comparisons (presented in Supplementary Materials: Figures S1–S3, respectively).

## 4. Conclusions

In this work, new cross-linked polymers were successfully synthesised from FMA and TO through free radical polymerisation followed by DA reaction. Participation of methacrylate groups in free radical polymerisation was confirmed by FTIR analysis, whereas the participation of furan moieties in DA reactions was confirmed by FTIR, Raman spectroscopy and TGA analysis. The presence of long hydrocarbon chains in TO helped to prevent the homopolymerisation of FMA molecules. The extent of polymerisation and the properties of the polymers depended on the stoichiometry of the monomers. With increasing tung oil content, the rate of reaction becomes higher than with the other materials containing less amounts of TO, resulting in brittle, uneven, non-homogeneous polymer systems. At room temperature, F50-T50-F showed storage modulus value of 540 MPa, which is 170% higher than those reported for other TO polymers. Furthermore, increasing tung oil content makes the chain movement easier in the system resulting in a lower *T*_g_ and a lower storage modulus of the polymer molecules. Thus, the thermo-mechanical and physical properties of these materials can be tuned by altering the ratios of raw materials to obtain wide variety of products from soft rubbery gel to brittle films. This kind of tung oil based polymers have found applications in the paint, lubricants and coating industry. Since the synthesised polymer is cross-linked in nature, it is difficult to process and further research is necessary by employing other polymerization techniques as well as controlling the molecular architecture to investigate suitable processability and to explore more application areas, where these polymers can serve as replacements to conventional petroleum based polymers.

## Figures and Tables

**Table 1:** Description of sample preparation and product yields.

Sample	FMA (wt %)	TO (wt %)	BPO (wt %)	Reaction Conditions	%Yield before Washing	%Yield after Washing
F50-T50	50	50	3	4 h at 85 °C, 2 h at 120 °C	97.3	89
F40-T60	40	60	3	4 h at 85 °C, 2 h at 120 °C	95.4	77
F30-T70	30	70	3	4 h at 85 °C, 2 h at 120 °C	79.8	45

FMA, TO and BPO denote furfuryl methacrylate, tung oil and benzoyl peroxide.

**Table 2:** Modulus values at room temperature and *T*_g_ from DMA (dynamic mechanical analysis) and different weight loss temperatures from TGA (thermogravimetric analysis).

Sample	Storage Modulus (MPa)	Loss Modulus (MPa)	*T*_g_ (°C) (From Tan Delta)	*T*_10_ (°C) (10% Weight Loss Temperature)	*T*_50_ (°C) (50% Weight Loss Temperature)
F50-T50-F	540	147	107	276	412
F40-T60-F	340	106	114	267	406
F30-T70-F	60	22	-	258	417
F50-T50	-	-	-	276	388
F40-T60	-	-	-	242	357
F30-T70	-	-	-	271	330

## Supplementary Materials

The following are available online at https://www.mdpi.com/2073-4360/12/2/258/s1, Figure S1. FTIR plots of free radically polymerised samples Polyfurfurylmethacrylate (PFMA), and FMA-TO polymers (F50-T50, F40-T60 and F30-T70). Figure S2. DSC plot of free radically polymerised Polyfurfurylmethacrylate (PFMA) (first heating, cooling and second heating scans are shown). Figure S3. TGA plot of free radically polymerised Polyfurfurylmethacrylate (PFMA) and FMA-TO polymers.
