# Supplementary material for: Synthesis and Properties of Thermosets from Tung Oil and Furfuryl Methacrylate
Source: Polymers (Basel). 2020 Jan 22;12(2):258. doi: 10.3390/polym12020258 (PMC7077211; doi:10.3390/polym12020258)
Supplement: Supplementary file 1 [file polymers-12-00258-s001.pdf]

polymers

Supplementary Materials

Synthesis and properties of thermosets from tung oil and furfuryl methacrylate

Sunanda Sain [1,]*, Dan Åkesson [1,]* and Mikael Skrifvars [1]

[1] Swedish Centre for Resource Recovery, University of Borås, SE-501 90 Borås, Sweden
* Correspondence: sunanda.sain@hb.se (S.S.); dan.akesson@hb.se (D.Å.)

FTIR plot

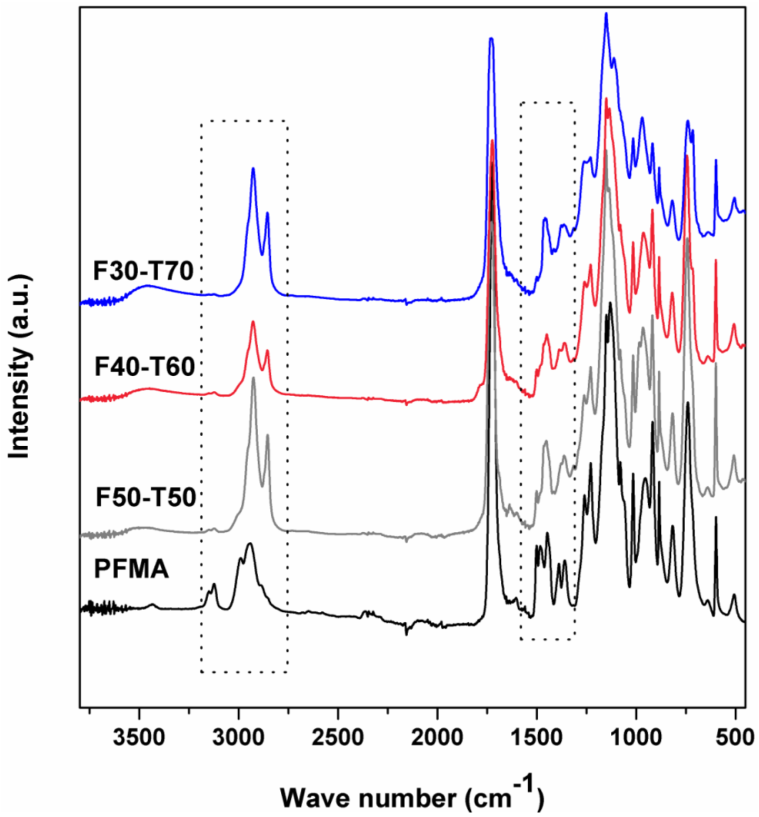

Figure S1. FTIR (Fourier-transform infrared) plots of free radically polymerised samples Polyfurfurylmethacrylate (PFMA), and FMA-TO (furfurylmethacrylate-tung oil) polymers (F50-T50, F40-T60 and F30-T70).

The differences in spectra between PFMA and FMA-TO polymers were observed in 1150–1700 cm^{-1} region and 2700–3500 cm^{-1} region (highlighted with black dotted square).

DSC plot

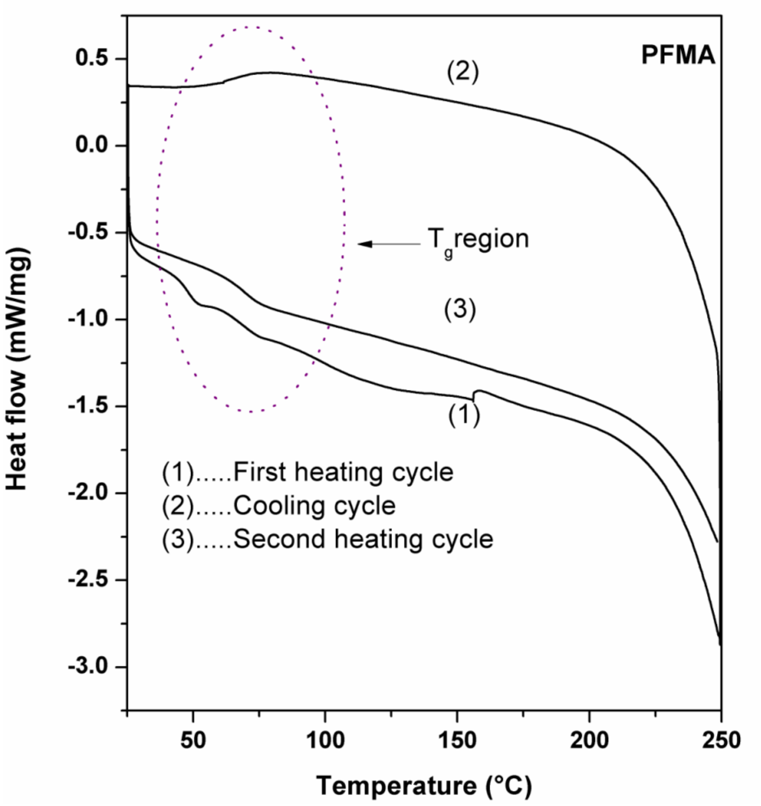

Figure S2. DSC (differential scanning calorimetry) plot of free radically polymerised Polyfurfurylmethacrylate (PFMA) [first heating, cooling and second heating scans are shown].

Glass transition temperature of free radically polymerized PFMA was found at 50–75 °C region. No exothermic transition has been happened at 140–170 °C in PFMA, whereas, sharp exothermic transitions have been observed at FMA-TO polymers at ~150 °C (graphs are in the main article).

TGA plot

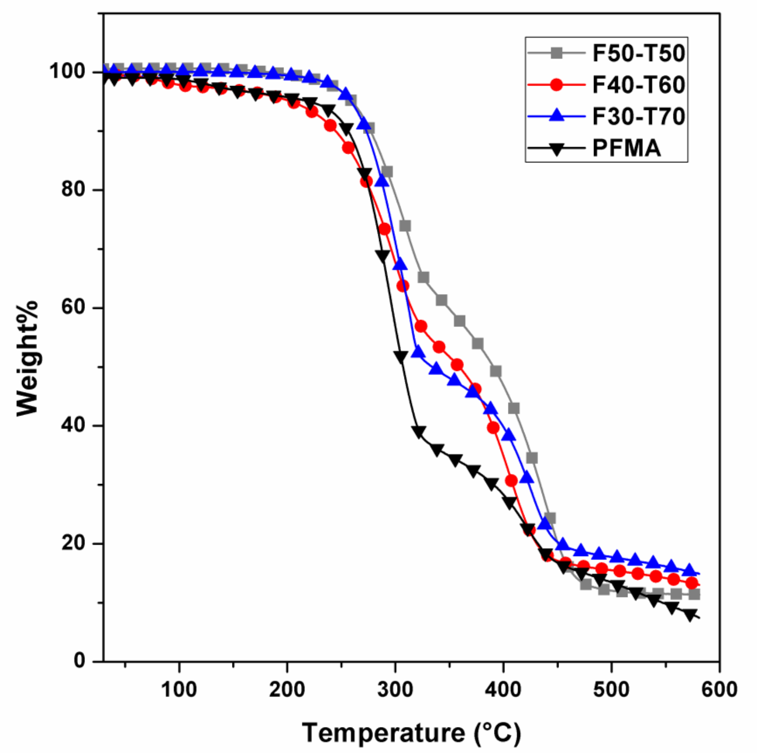

Figure S3. TGA (thermogravimetric analysis) plots of free radically polymerised Polyfurfurylmethacrylate (PFMA) and FMA-TO (furfurylmethacrylate-tung oil) polymers.

Thermal stability of PFMA was much lower than FMA-TO polymers.
